# Tracing Neurological Diseases in the Presymptomatic Phase: Insights From Neurofilament Light Chain

**DOI:** 10.3389/fnins.2021.672954

**Published:** 2021-05-24

**Authors:** Lorenzo Gaetani, Lucilla Parnetti, Paolo Calabresi, Massimiliano Di Filippo

**Affiliations:** ^1^Section of Neurology, Department of Medicine and Surgery, University of Perugia, Perugia, Italy; ^2^Neurology Unit, Fondazione Policlinico Universitario Agostino Gemelli IRCCS, Rome, Italy; ^3^Neuroscience Department, Università Cattolica del Sacro Cuore, Rome, Italy

**Keywords:** neurofilament light chain, multiple sclerosis, Alzheimer’s disease, frontotemporal lobar degeneration, amyotrophic lateral sclerosis, presymptomatic

## Abstract

The identification of neurological diseases in their presymptomatic phase will be a fundamental aim in the coming years. This step is necessary both to optimize early diagnostics and to verify the effectiveness of experimental disease modifying drugs in the early stages of diseases. Among the biomarkers that can detect neurological diseases already in their preclinical phase, neurofilament light chain (NfL) has given the most promising results. Recently, its measurement in serum has enabled the identification of neurodegeneration in diseases such as multiple sclerosis (MS) and Alzheimer’s disease (AD) up to 6–10 years before the onset of symptoms. Similar results have been obtained in conditions such as frontotemporal dementia (FTD) and amyotrophic lateral sclerosis (ALS), up to 2 years before clinical onset. Study of the longitudinal dynamics of serum NfL has also revealed interesting aspects of the pathophysiology of these diseases in the preclinical phase. This review sought to discuss these very recent findings on serum NfL in the presymptomatic phase of neurological diseases.

## Introduction

Reliable cerebrospinal fluid (CSF) and blood biomarkers allow for the *in vivo* measurement of axonal damage in neurological diseases. The detection of axonal damage has a variety of potentially useful experimental and clinical implications. Among them, it might serve as a sort of “alarm system” that can reveal the presence of a central nervous system (CNS) disease before its clinical onset. The identification of a neurological disease in its presymptomatic phase may have different benefits, including the expansion of the window of therapeutic opportunities. For such an “alarm system” to be efficient, the “sensor” of axonal damage has to be easily measurable, sensitive, reliable, and reproducible. In the case of a fluid biomarker, it should have robust evidence of validity, and it should be measurable through easy-to-perform longitudinal sampling.

Among axonal damage biomarkers, neurofilament light chain (NfL) is the best in terms of meeting these requirements (Gaetani et al., 2019). As a subunit of neurofilaments, it is released in CNS interstitial space upon axonal injury. From there, it reaches CSF and blood through dynamics that are still not completely understood (Gafson et al., 2020), but its concentrations in these biofluids are strongly correlated (Disanto et al., 2017).

Two enzyme-linked immunosorbent assays (ELISA) are currently available for measuring NfL in the CSF (Norgren et al., 2003; Gaetani et al., 2018), and ultrasensitive methods (such as electrochemiluminescence and single molecule array) have been developed to measure it at a lower concentration in blood (Rissin et al., 2010; Kuhle et al., 2015).

NfL has been demonstrated to reflect the severity of neurological diseases, as it would be expected from a biologically valid marker of axonal damage. From degenerative to inflammatory CNS diseases, NfL correlates with the severity of the clinical pictures in different functional systems, ranging from motor to cognition, and has clear predictive properties (Gaetani et al., 2019). The recent availability of assays that are able to measure NfL in the blood led to the investigation of its variations over time in individuals at high risk for neurological diseases. Studies have demonstrated that blood NfL can trace the presymptomatic course of different CNS disorders (Parnetti et al., 2019).

In this review, we summarize evidence on NfL in the presymptomatic phase of neurological diseases. For each disease, we will discuss its potential in implementing clinical research. Finally, we will provide insights on the contribution of NfL in understanding the early pathophysiology of CNS diseases.

## NfL in Presymptomatic Multiple Sclerosis

The natural history of multiple sclerosis (MS), the commonest chronic inflammatory disease of the CNS, is characterized by a long course, with clinical onset in early adulthood (Filippi et al., 2018). Both the possibility of a pediatric onset and the frequent detection at the time of diagnosis of inactive demyelinating lesions on brain magnetic resonance imaging (MRI) (Rovira et al., 2009) suggest that MS may have a long presymptomatic phase. Different disease-modifying drugs (DMDs) are available for MS treatment, and their efficacy often relies on an early diagnosis (Comi et al., 2017). Ideally, the detection of the disease in its presymptomatic phase would allow for the earliest treatment possible.

The potential of NfL in presymptomatic MS has been retrospectively tested in a case control study performed on United States military personnel who had serum samples stored in government repositories. Serum NfL was found to be higher in healthy soldiers who later developed MS compared to those who did not, with a median time between serum collection and MS onset of 6 years (range: 4–10). Of interest, the difference in serum NfL tended to increase as the onset of the disease approached, with a marked increase at the time of the first clinical manifestation. Intraindividual variations of serum NfL were also associated with a higher risk of subsequent MS development (Bjornevik et al., 2019). This suggests that the presymptomatic phase of MS can last up to 6 years and that, during this phase, serum NfL tends to increase, with a peak preceding the first clinical manifestation.

Similar findings have been found with CSF NfL in radiologically isolated syndrome (RIS), i.e., that condition characterized by the incidental finding of brain MRI abnormalities highly suggestive of MS in individuals who are asymptomatic or have non-MS specific symptoms (Okuda et al., 2009). RIS may be considered a sort of presymptomatic MS, given that 30–45% of these patients develop MS within around 2–5 years (Lebrun, 2015). In this specific group of patients, CSF NfL demonstrated to be an independent, though weak, predictor of the development of a first clinical episode suggestive of MS [hazard ratio (HR) = 1.02, 95% confidence interval (CI) 1.00–1.04, *p* = 0.019], or even of MS (HR = 1.03, 95% CI 1.01–1.05, *p* = 0.003). Higher CSF NfL values have also been associated with a shorter time to MS development (Matute-Blanch et al., 2018).

Taken together, these findings on blood and CSF NfL in presymptomatic MS may have several repercussions. On an experimental level, the possibility of verifying the effectiveness of pharmacological intervention in the presymptomatic phase of the disease requires a screening test to identify the subjects to be enrolled in a hypothetical clinical trial. The measurement of serum NfL could be that screening test, to be followed by more specific investigations, such as MRI and CSF analysis. Clinically, serum NfL could again serve as a screening test in individuals at high risk of developing MS, for example in people with a strong familiarity with the disease. These individuals could benefit from lifestyle interventions aimed at minimizing exposure to known environmental risk factors for MS. Additionally, a prodromal phase of MS lasting up to 5 years has been identified, and it is characterized by more frequent use of health care services for unspecific or minor symptoms in individuals who will later develop MS, compared to controls (Wijnands et al., 2017). It could therefore be hypothesized that, in subjects at high risk of MS and who begin to frequently use health care services for any reason, serum NfL could provide a screening tool to identify the presymptomatic or prodromal phase of the disease at an early stage.

## NfL in Presymptomatic Alzheimer’s Disease

Neurodegenerative diseases, though distinct from each other in terms of clinical manifestations, share different common features, such as the presence of a presymptomatic phase (Dickson et al., 2008; Eisen et al., 2014; Dubois et al., 2016). During this phase, the most characterizing pathophysiological mechanisms, e.g., amyloidosis and tauopathy in Alzheimer’s disease (AD)—the commonest neurodegenerative disease, have already taken place (Jack et al., 2013). As a consequence, neuronal loss begins before clinical symptoms, and remains below the clinical threshold the longer, the higher the neuronal functional reserve (Stern, 2012). In this presymptomatic phase, neuronal loss is still limited, and a therapeutic intervention could theoretically have the best chance to provide its maximum effectiveness. Once already in the clinical stages, potential DMDs could be ineffective or demonstrate a biological, but not clinical efficacy.

Another common aspect of neurodegenerative diseases is the presence of familial forms due to genetic mutations (Dion et al., 2009; Van Cauwenberghe et al., 2016; Kim and Alcalay, 2017). These familial forms share many pathophysiological and clinical features with the more common sporadic forms. Additionally, mutation carriers will develop the disease, often with a predictable age at onset, and therefore they represent a valid presymptomatic model of neurodegenerative diseases.

In a cross-sectional study performed on presymptomatic mutation carriers for familial forms of AD, namely carriers of pathogenic mutations in the genes coding for presenilin 1 (*PSEN1)* and amyloid precursor protein (*APP)*, serum NfL was found to be higher compared to non-carriers, suggesting ongoing quantifiable neurodegeneration already in the presymptomatic phase. Of interest, the mean estimated years from symptom onset was around 10 years, and serum NfL correlated with the distance with the estimated onset of the disease (Spearman ρ = 0.81, *p* > 0.0001). Specifically, individuals at a disease stage closer to the estimated clinical onset had higher NfL concentrations. Symptomatic carriers also had the highest value of serum NfL compared to presymptomatic carriers and non-carriers (Weston et al., 2017). In a similar cohort of patients with *PSEN1* and *APP* pathogenic mutations, serum NfL was confirmed to be significantly higher in mutation carriers compared to non-carriers up to 15 years before estimated symptom onset (Weston et al., 2019).

Of interest, when measuring serum NfL longitudinally, it has been found that the temporal dynamics of this biomarker differed between *PSEN1*, presenilin 2 (*PSEN2*), and *APP* mutation carriers and controls. The rate of change over time of serum NfL was able to discriminate between carriers and non-carriers a decade earlier than the single time-point measurement, i.e., around 16 years before the estimated onset of the disease. As noted in MS, in presymptomatic AD subjects, serum NfL peaked at the time of symptoms appearance, suggesting an acceleration in neuronal loss at the border zone between presymptomatic and symptomatic stages (Preische et al., 2019).

Taken together, these data suggest that AD has a long, gradually progressive presymptomatic phase that can be tracked by serum NfL changes over time. Therefore, the window for early detection of the risk of conversion from the asymptomatic to the clinical phase of AD might be particularly long and it should be longitudinally monitored. In AD, a marker of downstream neurodegeneration, such as NfL, might be useful as a tool for patients’ recruitment in clinical trials on presymptomatic subjects, as well as outcome measures to verify the potential in disease course modification along with the presymptomatic phase. However, the length of the preclinical phase of AD raises the complexity in the set-up of a clinical trial. In clinical practice, a blood test for the ongoing neuronal loss might be used to identify those individuals with subjective cognitive decline or minimal cognitive deficits to be prioritized for more detailed investigations in the suspicion of AD, such as CSF analysis or PET imaging for amyloid or tau-pathologies biomarkers.

## NfL in Presymptomatic Frontotemporal Dementia

Similar to other neurodegenerative diseases, frontotemporal dementia (FTD) may have familial forms, which are associated with mutations in the genes coding for progranulin (*GRN*), chromosome 9 open reading frame 72 (*C9orf72*), or microtubule-associated protein tau (*MAPT*) (Lashley et al., 2015). As for AD, these genetic forms represent a good model to understand the pathophysiology of the disease early in the presymptomatic phase.

In a multicenter cross-sectional study on symptomatic and presymptomatic carriers of *GRN, C9orf72*, and *MAPT* mutations, CSF and serum NfL were significantly higher in symptomatic compared to presymptomatic carriers, while no significant difference at the group level was found between presymptomatic carriers and controls (Meeter et al., 2016). In the same study, longitudinal CSF samples were available for five individuals, showing a three- to fourfold increase in NfL levels over conversion into the symptomatic stage in two mutation carriers who converted to manifest disease (Meeter et al., 2016). In line with this finding, in another study, the longitudinal rate of change of serum NfL was found to be similar between non-carriers and presymptomatic carriers, while it was significantly higher in converter patients, around 1–2 years before symptom onset (van der Ende et al., 2019). However, when modeled by age, a significant difference in serum NfL emerged between presymptomatic mutation carriers and non-carriers from the age of 48 years (van der Ende et al., 2019). This highlights the need for further longitudinal studies to better define the real extension of the preclinical phase of FTD-related neurodegeneration.

Overall, these preliminary results seem to suggest a shorter duration of the preclinical phase of FTD if compared with AD. Thus, the window for early detection of the risk of conversion from the asymptomatic to the clinical phase of FTD could be particularly short. This phase, therefore, should be closely monitored to identify those at-risk individuals who deserve more detailed investigations in the suspicion of FTD. A blood NfL increase in presymptomatic gene carriers could be a good biomarker to include these individuals in clinical trials, and a non-interventional study as preparation for pivotal clinical trials is ongoing, with the aim of qualifying blood NfL as an endpoint for the prevention of familial forms of FTD (ClinicalTrials.gov identifier: NCT04516499). Since the conversion to the symptomatic phase of FTD is shorter than in AD, clinical trials in these individuals might be easier to design.

## NfL in Presymptomatic Amyotrophic Lateral Sclerosis

As for AD and FTD, asymptomatic carriers of amyotrophic lateral sclerosis (ALS) gene mutations represent an opportunity to investigate the preclinical phase of the disease. The most frequent causative mutations involve the genes *C9orf72*, CuZn-superoxide dismutase (*SOD1*), fused in sarcoma (*FUS*), and TAR DNA binding protein (*TARDBP*) (Dion et al., 2009). Different studies on sporadic ALS have demonstrated that elevated CSF and serum NfL, as well as elevated CSF phosphorylated neurofilament heavy chain (pNfH)—a different neurofilament subunit, are valid markers of the neurodegeneration taking place in ALS with potential implications in the differential diagnosis (Oeckl et al., 2016; Steinacker et al., 2016). Over the last 5 years, more attention has been directed toward the preclinical phase of ALS, with findings that are in line with what was observed in other neurodegenerative diseases, especially for FTD.

CSF and serum NfL and CSF pNfH were initially found to be not significantly different between controls and asymptomatic gene mutation carriers, suggesting the possibility of a very short presymptomatic phase of the disease (Weydt et al., 2016). At the cross-sectional level, this finding was confirmed on independent populations (Benatar et al., 2018). However, when measuring serum NfL longitudinally, presymptomatic mutation carriers showed a progressive increase in serum NfL in contrast to the substantial stability of the biomarker over time in symptomatic ALS patients. Moreover, among individuals moving from the presymptomatic to the symptomatic phase, elevated serum NfL levels were observed as back as around 12 months before symptom onset with an increase lasting for the first 6 months after clinical conversion (Benatar et al., 2018). These results were obtained in a cohort of patients who, for the most part, were carriers of the *SOD1* mutation. When looking at the different mutations responsible for ALS, a longer presymptomatic phase was detected in *FUS* (2 years) and *C9orf72* (3.5 years) mutation carriers (Benatar et al., 2019).

Compared to other neurodegenerative diseases, ALS seems to have a short preclinical phase, especially in carriers of *SOD1* mutations, with serum NfL dynamics similar to that observed in FTD patients. These results open to the possibility of selecting asymptomatic patients at genetic risk for ALS who are most likely to develop manifest disease within a relatively short period of time. A presymptomatic increase in serum NfL might therefore become an eligibility criterion for a clinical trial on a potential DMD for ALS.

## Early Pathophysiology of CNS Diseases: the Lesson Learned From NfL Studies

**FIGURE 1:**
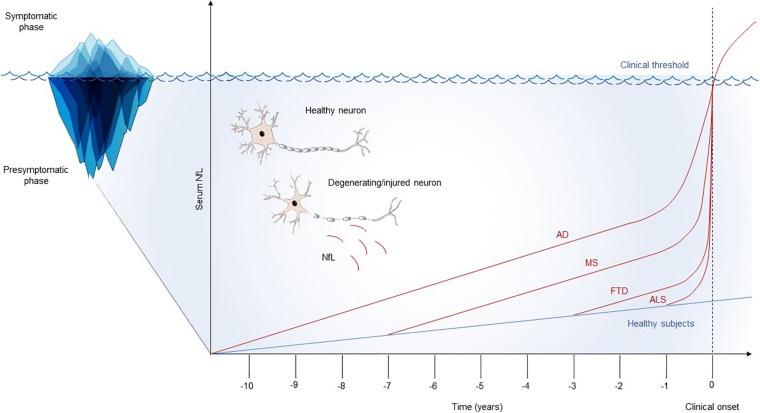
Schematic model of the longitudinal trajectories of serum NfL. Serum NfL studies highlight possible differences in the natural history of neurological diseases, with variable durations of the presymptomatic phase, probably reflecting the rate and overall burden of the underlying neurodegenerative processes. For all the diseases, an acceleration on serum NfL raise in proximity with the appearance of the first clinical manifestations has been documented. This longitudinal trend could suggest a flare in the pathophysiology of neurological diseases at the border zone between preclinical and clinical phases. AD, Alzheimer’s disease; ALS, amyotrophic lateral sclerosis; FTD, frontotemporal dementia; MS, multiple sclerosis; NfL, neurofilament light chain.

The study of the presymptomatic phase of neurological diseases represents a unique opportunity to understand the early pathophysiological mechanisms underlying neurodegeneration and neuroinflammation. Serum NfL has been shown to sensitively identify the neuronal damage that takes place in the presymptomatic phase of different neurological diseases. Interestingly, the duration of the preclinical phase could be a characteristic that depends on both the neurological reserve of the subject and the specific pathological processes taking place in the CNS (Figure 1). MS could have a long presymptomatic phase that can be caught by serum NfL up to 6 years before the clinical onset of the disease. Similarly, the presymptomatic stage of AD seems to be detected by serum NfL up to 10 years before clinical onset. On the contrary, diseases such as FTD and ALS could have a faster preclinical course, with a duration of the presymptomatic phase that can last around 2 years. This could be the consequence of faster neurodegenerative processes taking place in FTD and ALS compared to MS and AD. However, methodological differences dealing with the study designs should be considered when comparing the dynamics of NfL in the presymptomatic phase of different diseases. Further studies comparing CNS disorders with the same methodology are needed to confirm these preliminary findings.

Interesting insights on the transition from the presymptomatic to the symptomatic phase of neurological diseases have been provided by serum NfL studies. Indeed, its longitudinal dynamics seem to demonstrate that neurodegeneration progressively increases along time in the preclinical phases, with a fast acceleration as symptoms approached. The pathophysiological model that NfL trajectories suggest is that the transition of neurological diseases from the preclinical to the clinical phase is not only the consequence of progressive neuronal damage that, at a given time, exceeds the clinical threshold. In MS, AD, FTD, and ALS, the onset of the clinical picture is associated with a spike in the increase of serum NfL that suggests the possibility of a flare in the ongoing pathophysiology, which associates with the appearance of clinical symptoms.

This hypothesis deserves further investigation, such as a more detailed prospective analysis of the interaction between serum NfL and other pathophysiology-related biomarkers at multiple time points in the presymptomatic phase of neurological diseases. These studies might allow for a better understanding of the biological changes that take place in proximity with the appearance of the first clinical manifestations, hopefully identifying early therapeutic targets for neurodegenerative and neuroinflammatory diseases.

From a therapeutic point of view, the identification of neurodegeneration in the preclinical phase of CNS diseases could allow for the study of new and already established therapies before neurodegeneration flares up, with the goal of delaying or preventing the onset of clinically manifest disease in the population at risk for developing diseases such as MS, AD, FTD or ALS.

## Current Limitations of NfL and Future Directions

There are several limitations to the potential use of blood NfL as a biomarker in clinical practice. From an analytical point of view, reference materials must be standardized and the methods for NfL measurement in blood must be accessible to many laboratories. Another critical point is related to the lack of universal normative values. Recently, a population-based cohort study provided age-dependent cut-off levels for serum NfL, but the investigated population aged between 38 and 85 years and, therefore, information regarding younger adults was lacking. Additionally, for individuals aged > 60 years, a substantial variability of serum NfL was found, probably reflecting the contribution of subclinical brain tissue damage beyond the normal process of aging (Khalil et al., 2020). Furthermore, other potential sources of variability of blood NfL normal values (e.g., ethnicity) have not been considered yet. Population studies on multicenter cohorts are therefore needed both to verify the dynamics of serum NfL independent of CNS diseases and to have reliable normative values for all age groups.

Data obtained so far are at the group level and require deeper analysis and further longitudinal studies to interpret NfL concentrations at the individual level. Future studies should address the level of change in the concentration of NfL, which equates to a threshold change that can be accepted as clinically meaningful.

In the specific context of the use of serum NfL in the preclinical phase of neurological diseases, other limitations must be overcome. Data reviewed and discussed here have been retrospectively obtained for the most part. Prospective studies should confirm the estimated dynamics of this biomarker along the preclinical course of CNS diseases. Moreover, as far as neurodegenerative diseases are concerned, the data presented derive from studies on carriers of genetic mutations responsible for the familial forms of these diseases. Although genetic and sporadic forms share different common pathophysiological and clinical aspects, differences exist that require studies on serum NfL in presymptomatic sporadic neurodegenerative diseases. Such studies, however, are difficult to realize, given the need to include large populations of healthy individuals to be longitudinally followed-up for a long time. Additionally, blood NfL might temporarily increase because of acute CNS diseases, such as stroke, transient ischemic attack, and traumatic brain injury (Shahim et al., 2016; De Marchis et al., 2018), which could be a confounding factor in monitoring the trajectories of this biomarker. Therefore, longitudinal measurements of serum NfL should always be coupled with a thorough clinical assessment, and the exact timing for serum samplings in monitoring patients at risk for neurological diseases must be defined. Finally, the sensitivity of blood NfL in detecting CNS diseases even in the symptomatic phase spans between roughly 45% for MS (Sejbaek et al., 2019), to 80–90% for AD, FTD, and ALS (Lewczuk et al., 2018; Verde et al., 2018; Katisko et al., 2020), meaning that some symptomatic patients have NfL levels within the range of controls. Therefore, the use of blood NfL alone as a screening test could miss the remaining presymptomatic cases. Once overcome these issues, the cost-effectiveness of a screening test with longitudinal serum NfL measurements applied to large populations should be demonstrated.

## Conclusion

Serum NfL is an excellent tool to early detect neurodegeneration in CNS diseases and to investigate the dynamics of neuronal damage over time in their preclinical phase. These findings open to the possibility of improving patient selection in clinical trials to test the real disease-modifying potential of experimental therapies. The next studies in this field should focus on large and unselected cohorts of healthy individuals to be followed-up to the appearance of suspected clinical manifestation of CNS diseases. Converter patients should then undergo deeper investigations with other more disease-specific biomarkers to better characterize the transition from the submerged to the visible part of the iceberg of neurological diseases.

## Author Contributions

LG critically read, analyzed, and discussed the literature. LG and MDF conceived the outline of the manuscript and wrote the manuscript. PC and LP edited the manuscript and provided valuable discussion and criticism. All authors contributed to the article and approved the submitted version.

## Conflict of Interest

LG participated on advisory boards for and received writing honoraria and funding for traveling from Almirall, Biogen, Sanofi, Merck, Mylan, Novartis, Roche, and Teva. PC participated on advisory boards for and received funding for traveling, speaker honoraria, and research support from AbbVie, Biogen Idec, Merck, Genzyme, Novartis, Prexton, Teva, UCB, and Zambon. MDF participated on advisory boards for and received speaker or writing honoraria and funding for traveling from Bayer, Biogen Idec, Sanofi, Merck, Mylan, Novartis, Roche, and Teva. The remaining author declares that the research was conducted in the absence of any commercial or financial relationships that could be construed as a potential conflict of interest.
